# Panoptic Segmentation of Individual Pigs for Posture Recognition

**DOI:** 10.3390/s20133710

**Published:** 2020-07-02

**Authors:** Johannes Brünger, Maria Gentz, Imke Traulsen, Reinhard Koch

**Affiliations:** 1 Department of Computer Science, Kiel University, 24118 Kiel, Germany; rk@informatik.uni-kiel.de; 2 Department of Animal Sciences, Livestock Systems, Georg-August-University Göttingen, 37075 Göttingen, Germany; maria.gentz@uni-goettingen.de (M.G.); imke.traulsen@uni-goettingen.de (I.T.)

**Keywords:** computer vision, deep learning, image processing, pose estimation, animal detection, precision livestock

## Abstract

Behavioural research of pigs can be greatly simplified if automatic recognition systems are used. Systems based on computer vision in particular have the advantage that they allow an evaluation without affecting the normal behaviour of the animals. In recent years, methods based on deep learning have been introduced and have shown excellent results. Object and keypoint detector have frequently been used to detect individual animals. Despite promising results, bounding boxes and sparse keypoints do not trace the contours of the animals, resulting in a lot of information being lost. Therefore, this paper follows the relatively new approach of panoptic segmentation and aims at the pixel accurate segmentation of individual pigs. A framework consisting of a neural network for semantic segmentation as well as different network heads and postprocessing methods will be discussed. The method was tested on a data set of 1000 hand-labeled images created specifically for this experiment and achieves detection rates of around 95% (F1 score) despite disturbances such as occlusions and dirty lenses.

## 1. Introduction

There are many studies that show that the health and welfare of pigs in factory farming can be inferred from their behaviour. It is therefore extremely important to observe the behaviour of the animals in order to be able to intervene quickly if necessary. A good overview of the studies, the indicators found, and the possibility of automated monitoring is provided by [1]. Similarly, there are studies examining the various environmental factors (housing, litter, and enrichment) and how these factors affect behaviour [2,3,4].

Observing the behaviour of the animals over long periods of time cannot be done manually, so automated and sensor-based systems are usually used. Classical ear tags or collars can locate their position but have the disadvantage that the transmitter cannot provide information about the orientation of the remaining parts of the animal’s body. In addition, the sensor must be purchased and maintained for each individual animal. This is why computer vision is increasingly used, where the entire barn with all animals can be monitored with a few cameras. An overview of different applications with computer vision in the pig industry can be found in [5].

Based on 2D or 3D images, the position of the individual animals and their movements can be detected. From the positions alone, a lot of information can be extracted. By means of defined areas, the position can be used to identify, e.g., food or water intake [6]. Furthermore, interactions and aggression between the animals can be detected if they touch each other in certain ways (mounting and chasing) [7,8,9]. The behaviour of the entire group can also be evaluated. Certain patterns when lying down can reveal certain information about the temperature in the barn [10], or changes in positions over time can be converted into an activity index [11] or locomotion analysis [12].

**Figure 1:**
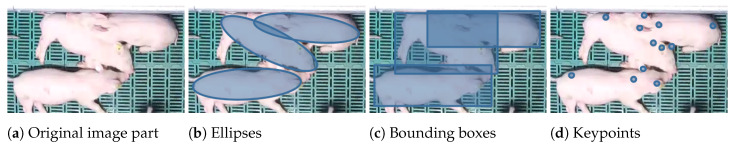
Visualization of different types of detection on an example image part (**a**); The proposed ellipses (**b**) provide more information about the pigs (like a weight-approximation) than the classic bounding boxes (large overlap) (**c**) or keypoints (**d**) where the affiliation to the individual animals has to be resolved afterwards.

Even though camera recording has many advantages due to its low-cost operation and noninvasive observation, the task of detecting animals reliably, even in poor lighting conditions and with contamination, is difficult. Previous work used classical image processing such as contrast enhancement and binary segmentation using thresholds or difference images to separate the animals from the background [6,9,13,14]. Later, the advantage of more sophisticated detection methods based on learned features or optimization procedures were presented [15,16]. With the recent discoveries in the field of deep learning, the detection of pigs with neural networks has also been addressed. The established object-detector networks were either applied directly to the pigs, or the detections found were postprocessed to visually separate touching pigs [17,18,19]. Although the detection rate with these object detection methods is very good, the resulting bounding boxes are suboptimal because, depending on the orientation of the animal, the bounding box may contain large areas of background or even parts of other animals (see Figure 1). Therefore, Psota et al. [20] proposed a method that avoids the use of bounding boxes and tries to directly detect the exact pose of the animal with keypoints on specific body parts (e.g., shoulder and back).

In this work, we close the gap between the too large bounding boxes and the sparse keypoints and try to identify the animals’ bodies down to the pixel level. We believe that the exact body outlines can help to classify the animals’ behaviour even better. The movement of individual animals can be depicted much better than with a bounding box, and the body circumference resulting from the segmentation can also be used to draw conclusions about the size and weight of the animals.

The main contribution of this thesis is the presentation of a versatile framework for different segmentation tasks on pigs together with the corresponding metrics.

The remainder of this work is organized as follows. In Section 2, the basic concepts of object detection based on bounding boxes, pixel-level segmentation, and key-points are listed. The proposed method is described in Section 3 followed by the evaluation in Section 4. The findings are discussed and concluded in Section 5 and Section 6.

## 2. Background

In recent years, methods based on neural networks have gained enormous importance in the field of image processing. Based on the good results in classification tasks, adapted network architectures were developed, which can also be used for the detection of objects [21,22]. The current generation of detection networks uses a combination of region proposals (bounding boxes) and classification parts, which evaluate the proposed regions [23,24,25]. With *Deepmask* [26,27] and *Mask-RCNN* [28], even object detectors have been shown which generate a pixel-level segmentation mask for each region found. Although these detectors provide very good results, the generated region proposals have the problem that only one object can be found at each position. This limitation is usually irrelevant because, in a projective image, each pixel is assigned to exactly one object anyway and two objects at the same position cannot be seen. However, if two elongated objects overlap orthogonally, the center of the objects may fall on the same pixel, which cannot be mapped by such a region proposal network.

Another area in which neural networks are very successful is (semantic) segmentation, in which each pixel is assigned a class (pixelwise classification) [29,30,31]. However, the classic semantic segmentation does not distinguish between individual objects but only assigns a class to each pixel. In order to separate the individual objects, an instance segmentation must be performed. For this purpose, the semantic segmentations are extended, for example, such that the output of the network is position-sensitive in order to identify the object boundaries [32]. Another solution is to count and recognize the animals in a recursive way. For this purpose, one object after the other is segmented and stored until no more objects can be found [33,34]. Since the networks are designed to predict certain classes, the classes can also be chosen to help distinguish the instances. Uhrig et al. [35], for example, use the classes to encode the direction to the center of the corresponding object for each pixel. Since the direction to the center of the object is naturally different at object boundaries, the individual instances can be separated. To assign the pixels to individual instances, a high-dimensional embedding can also be used. As described by De Brabandere et al. [36], a high-dimensional feature space is formed, and for each pixel in the image, the network predicts the position in space. Via discriminative loss, pixels belonging to the same object are pushed together in the embedding space and pixel clusters of different objects are pushed apart. With a subsequent clustering operation, the instances in the embedding can then be separated. The relatively new definition of *panoptic segmentation* [37] defines the instance segmentation as a combination of *semantic segmentation* and *instance segmentation*. This combined approach has also been directly implemented recently [38,39,40]. However, the output of an object detector is often used as a reference, so these approaches probably also have problems when objects share the same position in space.

Another approach for the segmentation of individual instances is the detection of certain key points, which are then meaningfully combined into the individual instances using skeleton models [41,42,43].

As described in the introduction, detection with bounding boxes and detection via key points has already been demonstrated on pigs. This work follows the definition of *panoptic segmentation* and aims at the pixel accurate segmentation of individual pigs based on pixel embedding as described in [36].

## 3. Proposed Method

**Figure 2:**
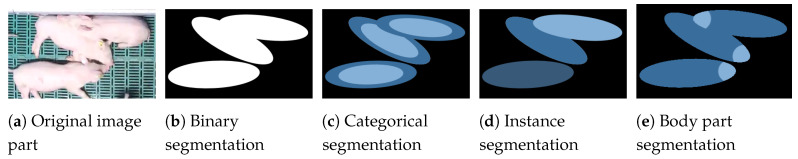
Visualization of the different experiments presented in this work: (**a**) original image; (**b**) The binary segmentation distinguishes only between foreground and background; A categorical segmentation can be used to separate the individual animals (**c**) or to classify body parts (**e**); (**d**) The network is trained to directly tell the affiliation of the pixels to the individual animals.

The goal of the proposed method is a *panoptic segmentation* [37] of all pigs in images of a downward-facing camera mounted above the pen. *Panoptic segmentation* is defined as a combination of *semantic segmentation* (assigning a class label to each pixel) and *instance segmentation* (detecting and segmenting each object instance). Therefore, the semantic segmentation part differentiates between the two classes background and pig, whereby the instance segmentation part is used to distinguish the individual pigs (see Figure 2b,d).

The proposed method for the *panoptic segmentation* is an extension of classical semantic segmentation. Therefore, in this paper, the complexity of segmentation is increased step by step, resulting in four separate experiments. First, a simple binary segmentation is tested (see Figure 2b). In the second experiment, the individual animals are extracted from a semantic (or categorical) segmentation (see Figure 2c). The third experiment shows a pixel precise instance segmentation based on a combination of the binary segmentation and pixel embedding. The learned pixel embedding serves as input for a clustering postprocessing step, which groups the pixels belonging to the individual animals (see Figure 2d). In the last experiment, the embedding is combined with a body part segmentation (see Figure 2e), which adds an orientation recognition to the instance segmentation.

**Figure 3:**
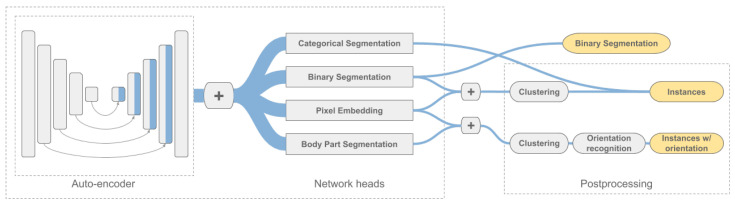
Schematic representation of the proposed framework. The auto-encoder is an U-Net architecture (depiction adopted from [44]). The individual stages consist of several blocks, each with several layers. Scaling down or up is done between the stages. Skip connections are used to combine the results of the encoder and decoder stages. The network is equipped with different heads for the different experiments. The output is processed afterwards to yield the desired results.

All experiments are based on the same network architecture. Only the last layers are adjusted to obtain the required output. This way, the presented framework can be easily adapted to each of the experiments. An overview of the framework is given in Figure 3. As described above, the inputs are fed through the network with one or more of the different heads, depending on the experiment. Then the outputs are combined if necessary and in a post-processing step the instances are extracted as ellipses.

### 3.1. Representation of the Pigs

In order to perform *panoptical segmentation* instance by instance and as accurately as possible, manual annotation should contain such instance information with pixel accuracy. Since the choice of annotation method always requires a trade-off between effort and accuracy, a pixel accurate annotation is preferable but also very costly. In contrast, bounding boxes can be drawn quickly but would contain large background areas in addition to the marked pig, especially if the pig is standing diagonally to the image axes (see Figure 1c). Based on existing work [6,10,13], ellipses were therefore chosen as annotations. They are also very easy to draw (due to the two main axes) and adequately reproduce the pigs’ bodies on the images of a downward facing camera. Except for small mistakes (e.g., when the animal turns its head to the side), the pixels belonging to the individual animals can thus be easily captured. Since the area of the ellipses correlates approximately with the volume of the animals, the ellipses have the further advantage of allowing conclusions to be drawn about the volume and the weight of the animals. Although pixel accurate segmentation was the objective, the network can of course only learn segmentation based on the annotated ellipses. However, as shown in Section 4.5, the network adapts to the contours of the animals rather than strictly following the elliptical shape. Therefore, the advantage of simple annotation outweighs the slight loss of accuracy. Of course, all subsequent steps could also be performed on the segmentation label generated on the basis of the annotated ellipses. In this paper, however, ellipses were deliberately extracted from the segmentation produced by the network to simplify storage and processing for later steps. For subsequent tracking or position evaluations, the five parameters per ellipse are completely sufficient, so there is no need to store the complete segmentation. By aligning the ellipse (first main axis), the orientation of the animals is also stored during manual annotation. If animals overlap, the order in which the pixels in the label image are drawn must correspond to the reversed order of the animals in the camera’s visual axis. This ensures that the pixels of the animals on top overwrite the pixels of the covered animals (see Figure 2b,d). Therefore, the order with respect to the camera’s visual axis is also included in the annotation.

**Figure 4:**
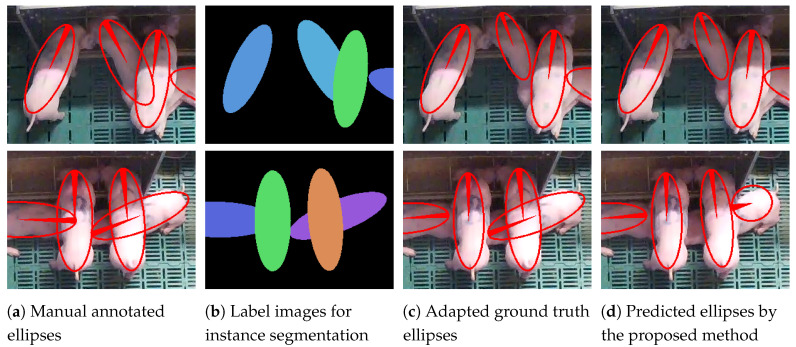
Two examples of the manually annotated ellipses (**a**); the label images created for instance segmentation (**b**); the extracted ground truth ellipses (**c**) and the results (**d**). The filled part of the ellipses shows the identified orientation of the animals. Note the adjusted overlaps in (**c**), which allow a comparison with the predicted ellipses. The lower picture in (**d**) shows a faulty detection.

The ellipses must be able to overlap in the manual annotation to capture all pixels belonging to an animal (see Figure 4a). While depth sorting, described above, ensures that each pixel is uniquely assigned to a single animal (see Figure 4b), the pixel-level segmentations cannot be compared to the originally annotated ground truth ellipses anymore. If the animals overlap, the annotated ellipses and the found segmentations differ in size. In order to generate comparable data for the evaluation, new ellipses were extracted from the generated label images by fitting ellipses into the segmentations (see Figure 4a–c). These adapted ground truth ellipses are used to compare the ellipses extracted from the segmentation output of the networks to the manual annotations.

### 3.2. Network Architecture

The typical network architecture for semantic segmentation consists of an encoder and a decoder part. The encoder transforms the input image into a low-dimensional representation, which the decoder then converts into the desired output representation (this combination is often also called auto-encoder). The encoder is structured similarly to a classification network, whereas the decoder is a combination of layers symmetrical to the encoder but with upsampling steps instead of the downsampling steps. To further improve the segmentation results, skip connections are often added. To make the information from the different resolution levels usable, these connections merge the intermediate results of the encoder with the corresponding upsampling levels in the decoder. Well-known versions of such networks are for example U-Net [30] and LinkNet [45]. Another approach to use the information from the different downsampling layers from the encoder aiming at obtaining dense segmentation in the original resolution is called Feature Pyramid Network (FPN) [46]. The predictions in this approach are computed at different scales and merged afterwards. In a similar way, the Pyramid Scene Parsing Network (PSPN) [47] uses a pyramid pooling module to access extracted features at different scales. To capture objects and background at different scales, the DeepLab network family [31,48,49,50] also uses pyramid pooling but combined with dilated convolutions. The latest version DeepLabv3+ [50] uses the predecessor DeepLabv3 [31] as an encoder and adds a special decoder. With this setup, the network achieves state-of-the-art results. Image segmentation can also be improved by attention modules. This allows networks to weigh features from previous layers and to thus improve the emphasis and gathering of information. Attention can be combined with pyramids [51], or different attention modules work in parallel [52] or one after the other [53]. A good overview of the latest developments in network architectures for semantic segmentation is provided in [54].

A U-Net with different encoder architectures was used in this work because it gave the most stable results on the data set used. However, the presented framework can also be combined with any other network architecture for image segmentation. To do so, the auto-encoder in the pipeline would simply be replaced (see Figure 3). More details on the implemented architecture can be found in Section 4.4. In Section 4.8, an ablation study evaluates additional backbones and hyperparameters. The possibility of using a different network architecture is also discussed.

### 3.3. Binary Segmentation

A binary segmentation is the basis for many of the classical approaches to pig detection [6,10,13,14]. At the same time, it is a comparably simple task for a neural network. Once solved, however, foreground segmentation can also be used to simplify more complex procedures, e.g., to apply them only to the important areas of the image (see Section 3.5).

For the binary segmentation, the network learns which pixels belong to the pigs and which belong to the background. Therefore, for each pixel $x_{i}$, it predicts a probability $p(x_{i})$ to which the pixel belongs to a pig (with the corresponding opposite probability $\left(1-p\left(x_{i}\right)\right)$ that the pixel belongs to the background). The training data consist of binary label images based on the manually annotated ellipses (see Figure 2b), where each pixel in the label image is a binary variable $y_{i}$, indicating whether the pixel belongs to the background (value 0) or to a pig (value 1).

The network is set up with the architecture described in Section 3.2 but with only one output layer. The output has the same spatial dimension as the input but with only one channel and a sigmoid activation function that generates the probability estimate for each pixel. The loss function is the cross-entropy loss:

(1) $$L=-\frac{1}{N}\sum_{i=1}^{N}y_{i}\;\cdot \;\log(p\left(x_{i}\right)+\left(1-y_{i}\right)\;\cdot \;\log\left(1-p\left(x_{i}\right)\right)$$

During inference, the predicted probability values are thresholded to create the final binary segmentation.

### 3.4. Categorical Segmentation

In the second experiment, a semantic or categorical segmentation is applied to be able to separate the individual instances. Based on the direction-based classes described in Uhrig et al. [35], the semantic segmentation is set up with the classes *background*, *outer edge of an animal*, and *inner core of an animal* (see Figure 2c) to recognize the outer boundaries of the animals. In other words, it defines a distance-based classification which encodes the distance to the pigs center in discrete steps, whereby the inner-core area is just a scaled down version of the original manually annotated ellipse. With these three classes, the training data are categorical label-images with an one-hot vector $t_{i}$ at each pixel, indicating one positive class and two negative classes.

In the existing network architecture, only the last layer is adapted such that the number of channels corresponds to the number of classes (C=3) defined in the experiment. Since each pixel can only belong to one of the *C* classes, the vector $x_{i}$ along the channel axis at each pixel location is interpreted as a probability distribution over the *C* classes. Such a probability distribution can be generated with the softmax activation function on the output layer. The loss function is the categorical cross-entropy loss over all *N* pixels and the *C* classes:

(2) $$L=-\frac{1}{N}\sum_{i=1}^{N}\sum_{j=1}^{C}t_{i,j}\;\cdot \;\log\left(x_{i,j}\right)$$

While in the binary segmentation the individual instances blend when they overlap, the centers of the animals and thus the individual instances can still be reconstructed with this method. A detailed description of the extraction process follows in Section 4.3.

### 3.5. Instance Segmentation

**Figure 5:**
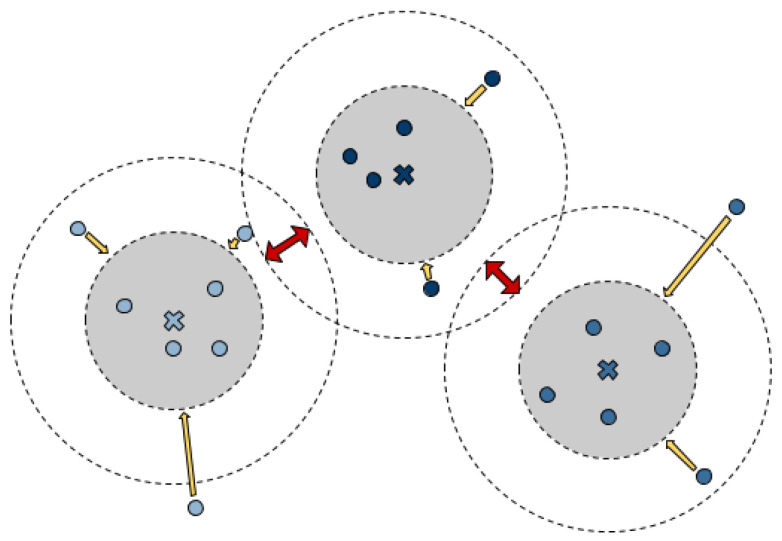
Illustration of the forces acting on the pixels to form the clusters (image adopted from [36]): With the variance term (yellow arrows), the pixels are drawn in the direction of the cluster mean (crosses). The distance term (red arrows) pushes the different clusters apart. Both forces are only active as long as the threshold values are not reached (inner circle for the cluster variance and outer circle for the distance).

Categorical segmentation is a rather naive approach, where the boundaries should prevent the individual animals from blending together. Therefore, in the third experiment, each pixel in the image should be assigned to a specific animal (or the background). For this task, De Brabandere et al. [36] have introduced a discriminating loss function which uses a high dimensional feature space in which the pixels of the input image are projected in (pixel embedding). The network learns to place the pixels belonging to one object in this space as closely together as possible, while pixels belonging to other objects are placed as far away as possible (see Figure 5).

The loss function is a weighted combination of three terms, which act based on the individual instances given by the annotated data:

1. **Variance term** The variance term penalizes the spatial variance of the pixel embeddings belonging to the same instance. For all pixels that belong to the object (according to the annotated data), the mean is calculated, and then for all object pixels, the distance to the mean is evaluated. This forces the points in the feature space to cluster.
2. **Distance term** The distance term keeps the calculated means of the clusters at a distance.
3. **Regularization term** The regularization term keeps the expansion of all points in the feature space within limits and prevents them from drifting apart.

Following the definition from [36], for each training example, there are *C* objects (or classes) to segment (the pigs plus the background). $N_{c}$ is the number of pixels covering object *c*, and $x_{i}$ is one pixel embedding in the feature space. For each object *c*, there is a mean of all its pixel embeddings $\mu_{c}$. ∥·∥ is the L1 norm. In addition, the loss is hinged to be less constrained in the representation. The pixel embeddings of the objects do not need to converge to exactly one point but should reach a distance below a threshold $\delta_{v}$. In the same way, the distance between two different mean embeddings must only be greater than or equal to the threshold $\delta_{d}$. This is mapped with the hinge-function ${\left[x\right]}_{+}=max\left(0,x\right)$. Now the three terms can be formally defined as follows:

(3) $$L_{reg}=\frac{1}{C}\sum_{c=1}^{C}∥\mu_{c}∥$$

(4) $$L_{var}=\frac{1}{C}\sum_{c=1}^{C}\frac{1}{N_{c}}\sum_{i=1}^{N_{c}}{\left[∥\mu_{c}-x_{i}∥-\delta_{v}\right]}_{+}^{2}$$

(5) $$L_{dist}=\frac{1}{C(C-1)}\underset{c_{A}\neq c_{B}}{\sum_{c_{A}=1}^{C}\sum_{c_{B}=1}^{C}}{\left[2\delta_{d}-∥\mu_{c_{A}}-\mu_{c_{B}}∥\right]}_{+}^{2}$$

The final loss function *L* with weights α,β and γ is given as follows:

(6) $$L=\alpha \;\cdot \;L_{var}+\beta \;\cdot \;L_{dist}+\gamma \;\cdot \;L_{reg}$$

#### 3.5.1. Postprocessing

After the network has been used to create the pixel embedding on an input image, the individual instances must be extracted from it. De Brabandere et al. [36] propose the use of the mean-shift algorithm to identify cluster centers and afterwards to assign all pixels belonging to the cluster (in terms of the $\delta_{v}$ threshold) to the same object. In this work, the hierarchical clustering algorithm HDBSCAN [55] is used instead, as it shows improved performance in high-dimensional embedding spaces. HDBSCAN is density based hierarchical clustering and therefore optimally suited for the required clustering. It starts with a thinning of the non-dense areas. Then, the dense areas are linked to a tree, which is converted into a hierarchy of linked components. Thus, a condensed cluster tree can be created by the parameter of minimum cluster size, and from this tree, the final flat clusters can be extracted.

#### 3.5.2. Combined Segmentation

**Figure 6:**
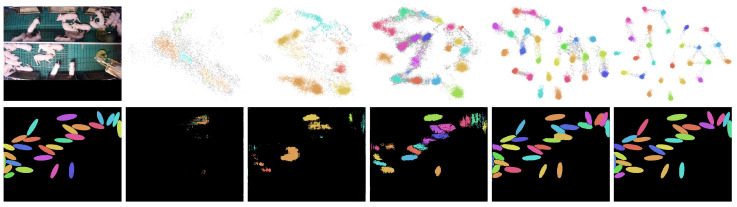
Results of the combined segmentation. On the left the original input image and below the ground truth label are shown. The top row depicts a two-dimensional embedding space. The bottom row depicts the corresponding binary segmentation and the assignment of the clusters. The snapshots are created after 1, 2, 3, 10 and 80 gradient updates. The network was trained solely on the shown input image to generate the results shown here for illustration purposes.

Since each pixel is mapped in the embedding, there are many data points that have to be clustered. At normal HD camera resolutions, this quickly adds up to a million data points. To accelerate clustering, a combined solution of discriminating and binary segmentation was designed. With the binary segmentation, a mask is created that contains only the pixels that belong to the animals. Thus, only those pixels are fed into the clustering process that are relevant for the differentiation of the individual animals. Figure 6 shows an example of the distribution of pixels in a two-dimensional embedding and the clustering applied to the binary segmentation.

The network architecture only needs to be adapted slightly, since the architectures of the two experiments only differ in the last layer. In order to generate both outputs simultaneously, the network is equipped with two heads, which generate the corresponding outputs from the outputs of the autoencoder. The two heads are trained with the appropriate loss functions and feed the gradient updates equally weighted into the auto-encoder network.

### 3.6. Orientation Recognition

If the pixel segmentation approximates to an ellipse shape, the major axis of the final extracted ellipse will match the orientation of the animal. However, since ellipses are symmetrical with a rotation of 180 degrees, the orientation of the animals can only be detected correctly up to this 180 degree ambiguity. Since the correct orientation was captured during manual annotation, this ambiguity can also be resolved. Therefore, the combined method described in the previous section uses a categorical segmentation with the classes *background*, *body*, and *head* instead of a binary segmentation (see Figure 2e). In postprocessing, the classes then can be used to determine the orientation of the animals as described in Section 4.3.

## 4. Experimental Results

### 4.1. Dataset

The data used in this work was obtained from a previous study on the influence of different rearing systems on pig welfare: Influence of different farrowing and rearing systems on animal welfare, animal health and economy in pig production (Inno-Pig) funded by the Federal Office for Agriculture and Food of Germany and the Landwirtschaftliche Rentenbank (project no. 2817205413 and 758914). The pictures were taken in a research facility that meets the standards of conventional pig rearing. The authors declare that the experiments were carried out strictly following international animal welfare guidelines (in consultation with the animal welfare officer of the Chamber of Agrigulture Schleswig-Holstein, Rendsburg, Germany).

Five cameras were installed, with each camera covering two 5.69 m${}^{2}$ pens, each with a maximum of 13 animals. The animals were housed at the age of 27 days and remained in the facility for 40 days. The recordings of this data set covered a period of four months. From all available videos, 1000 frames with a resolution of 1280 × 800 pixels were randomly selected and manually annotated. The images from one of the five cameras were declared as a test set, so that the evaluation is based on images of pens that the network never saw during the training. The images of the remaining four cameras make up the training and validation set. The data sets contain normal color images from the daytime periods and night vision images with active infrared illumination from the night periods. In addition, the cameras occasionally switched to night vision mode during the day due to dirty sensors. In the evaluation, however, a distinction is only made between color images and active night vision regardless of the time of day. An overview can be found in Table 1.

**Figure 7:**
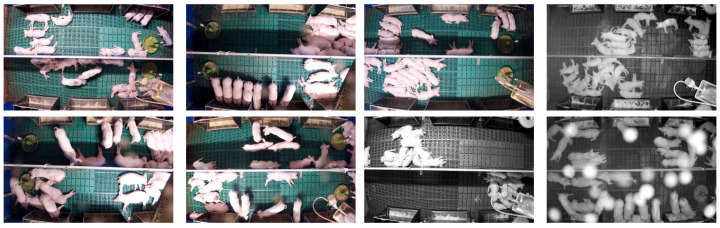
Some sample images from the data set used: Note the poor lighting conditions, the dense grouping of the animals, and the distortions during active night vision caused by dirt on the lens.

In Figure 7, some example images from the data set are shown. Some of the challenges of working in pigsties can be clearly seen. For one thing, the camera position cannot always be chosen optimally so that occlusions cannot be avoided. Furthermore, the lighting and the natural incidence of light cannot be controlled, so the exposure conditions are sometimes difficult. Last but not least, the cameras get dirty over time, resulting in disturbances and malfunctions (such as the erroneously active night vision).

### 4.2. Evaluation Metrics

For the task of *panoptic segmentation*, Kirillov et al. [37] also proposed a metric called *panoptic quality* (PQ). It is very similar to the well-known F1 score but takes into account the special characteristic that each pixel can only be assigned to exactly one object. It first matches the predicted segments with the ground truth segments and afterwards calculates a score based on the matches.

Since each pixel can only be assigned to one object, the predicted segments cannot overlap. Therefore, it can be shown that there can be at most one predicted segment for each ground truth segment, with an intersection over union (IoU) of strictly greater than 0.5 [37]. Each ground truth segment for which there is such a matching predicted segment counts as a *true positive* (TP). Predicted segments that do not sufficiently overlap any ground truth segment count as *false positives* (FP), and uncovered ground truth segments count as *false negatives* (FN). For all the predicted segments *p* and the ground truth segments *g*, PQ is defined as follows:

(7) $$PQ=\frac{\sum_{(p,g)\in TP}IoU\left(p,g\right)}{\left|TP\right|+\frac{1}{2}\left|FP\right|+\frac{1}{2}\left|FN\right|}$$

For better comparability with other work, the F1 score, precision, and recall are also evaluated in the experiments (see Section 4.5). F1, precision, and recall are based on the same TP, FP, and FN as the PQ.

### 4.3. Ellipse Extraction

As described in Section 3.1, the detected poses should be stored as ellipses. Therefore, the individual ellipses are extracted from the network outputs in a subsequent step. For categorical segmentation, all pixels of the class *inner core of an animal* (see Section 3.4) are searched first using a blob search. The individual separate blobs are then interpreted as individual animals. For this, an ellipse is fitted to the segmented pixel with the algorithm of Fitzgibbon [56]. Since the core of an animal was generated from the scaled-down version of the manually annotated ellipse, the ellipse adapted from the blob can then simply be scaled up accordingly.

When using the segmentation with the discriminative loss and the clustering, the ellipses can simply be fitted to the pixels of the individual clusters after backprojecting the pixels from the embedding into image-space. As described in Section 3.5, the binary mask of the combined approach is used here to process only the pixels that belong to the animals while masking out the background. If the orientation of the animals is also detected, the classes *body* and *head* can be combined to achieve the binary segmentation. Once the ellipses are fitted, the original categorical segmentation can be used to identify the side of the ellipse where the head was detected.

Depending on the complexity of this postprocessing, the runtime of the entire process changes. The exact runtimes are therefore given in Section 4.6.

### 4.4. Implementation Details

As described in Section 3.2, a U-Net was used, as its modular design allows the use of different classification architectures as encoders. Thus, it is possible to benefit from the latest developments in this field. With ResNet34 [57] and Inception-ResNet-v2 [58], two established classification networks were used as encoder backbones. They both consist of single blocks that combine different convolution operations with a shortcut connection. With these shortcut connections, the optimizer does not have to learn the underlying mapping of the data but simply a residual function [57]. The blocks are organized in different stages, and each stage is followed by a downscaling. The decoder part imitates the stages but uses an upscaling layer instead of downscaling. Via the skip connections, the stages of the encoder are connected to the stages of the decoder where they are combined with the results of the encoder (see the auto-encoder in Figure 3). Exact details on the structure of the blocks in the encoder backbones can be found in the corresponding papers.

The network was implemented with the *segmentation models* library [44]. For all experiments, the *Adam*-Optimizer [59] with an initial learning rate of 0.00001 was used.

To speed up the calculation of the network and any subsequent clustering, the images were scaled down to a resolution of 640 × 512 pixels. Additionally, the training images were augmented during the training with the *imgaug* library [60] to achieve a better generalization. The augmentation included different distortions, affine transformations, and color changes (e.g., grayscale to simulate active infrared illumination) and increased the amount of training images by a factor of 10. For all the image-related pre- and postprocessing tasks (such as the ellipse fitting), the OpenCV-library [61] was used.

For pixel embedding, an eight-dimensional space was used. The thresholds in the discriminative loss in Equations (4) and (5) were set to $\delta_{v}=0.1$ and $\delta_{d}=1.5$. The weights in the final loss term in Equation (6) were set to α=β=1.0 and γ=0.001. The values were taken from the original paper [36], except for the threshold $\delta_{v}$, which was decreased to improve the density-based clustering. For clustering, the HDBSCAN implementation from McInnes et al. [62] was used with the minimal cluster size set to 100. The influence of these hyperparameters is evaluated in the ablation studies in Section 4.8.

### 4.5. Evaluation

In order to evaluate the methods described in Section 3, they were all run on the test data set. To investigate the influence of different backbones, all experiments were performed with both backbones. A distinction was also made between day and night vision images to test the robustness of the methods.

#### 4.5.1. Binary Segmentation

In binary segmentation, the network predicts a probability that a particular pixel belongs to a pig or the background. This probability is converted into a final decision using a threshold value of 0.5. The binary pixel values can then be compared with the ground truth images using the Jaccard index. The accuracy results of the binary segmentation are listed in Table 2.

**Figure 8:**
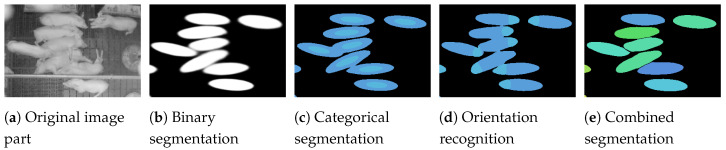
Results from the different experiments on an example image (cropped) (**a**); Depicted are the simple binary segmentation (**b**); the categorical segmentation with classes *outer edge of an animal* and *inner core of an animal* (**c**); the body part segmentation for the orientation recognition with classes *head* and *rest of the body* (**d**) and the combined segmentation with the results of the clustering, masked with the binary segmentation (**e**).

The ellipses cover the body of the animals only approximately (see Section 3.1). Therefore, the network sometimes receives ambiguous information, where pixels that can be clearly recognized as background still have the label *pig*. The network produces mainly elliptical predictions, but the segmented areas also follow the body of the animals (see Figure 8b). Since the label images only contain undistorted ellipses, an accuracy of 100% is never achievable for the network.

#### 4.5.2. Categorical Segmentation

For categorical segmentation, the class *inner core of an animal* was set to 50% of the size of the ellipses (see Figure 8c). The results are shown in the upper part of Table 3. Besides the accuracy of the categorical segmentation (again measured with the Jaccard index), now also the extracted ellipses (see Section 4.3) were compared to the manually annotated ellipses using the *panoptic quality* metric. F1 score, precision, and recall are listed in detail in Table 8.

#### 4.5.3. Instance Segmentation

For this experiment, a combined network was trained to predict the association of each pixel with the individual animals in an eight-dimensional space together with the binary segmentation. The results are shown in the lower part of Table 3. F1 score, precision, and recall are listed in the lower part of Table 8. It is important to note that the combined processing of pixel embedding and binary segmentation in a shared backbone does not affect the accuracy of the binary segmentation. Therefore, a synergy effect of the two tasks can be assumed.

#### 4.5.4. Orientation Recognition

For orientation recognition, the same combined network as before was used but the binary segmentation was replaced with body part segmentation (see Figure 8d). The orientation of the ellipses is reconstructed as described in Section 4.3. To evaluate the accuracy of the orientation recognition, the orientation of all correctly identified pigs (true positives) was assessed over the complete test set. The results are summarized in Table 4. Although a categorical segmentation is now applied instead of binary segmentation, a comparison with the values in Table 3 shows that the accuracy of ellipse detection is not affected.

### 4.6. Runtime Evaluation

Due to the already efficient implementation of the neural networks and the use of suitable hardware (e.g., GPUs), the runtime of the inference of the networks can hardly be improved. In the different experiments, the networks differ only in the last layers, so that the postprocessing steps for ellipse extraction (see Section 4.3) have decisive influence on the runtime. To show these differences, the runtime of the individual components was assessed during the evaluation of the test set and summarized in Table 5. The evaluation was performed on a desktop PC with an Intel i7-6700K CPU @ 4.00GHz CPU and a NVIDIA GeForce GTX TITAN X GPU.

With approx. 35 ms, the categorical version can be evaluated completely in real-time (24 fps) with the ResNet34 backbone. With the much more complex Inception-ResNet-v2 backbone, still more than 10 frames per second can be processed, which is one of the common recording rates for surveillance video. In the combined approach, clustering alone is responsible for a large part of the postprocessing runtime, with over two seconds. This approach is therefore completely unsuitable for online processing of video data.

### 4.7. Cross-Validation

To further prove the robustness of the proposed method, a cross-validation was performed on the five cameras contained in the data set. In each run, one of the cameras was declared as the test set and the images from the four remaining cameras were used to provide the training and validation set.

The values listed in Table 6 confirm the results shown in the evaluation for all runs of the cross-validation. For all metrics, the standard deviation is very small.

### 4.8. Ablation Studies

The experiments conducted with the two differently complex encoder architectures already suggest that the influence of the backbone is marginal. Nevertheless, additional experiments were carried out to confirm this assumption. To increase the speed of the tests, the resolution of the input images was further reduced to 320 × 256 pixels. The results are summarized in Table 7.

#### 4.8.1. Classification Backbone

As described in Section 3.2, the chosen U-Net architecture can be set up with different classification backbones. In addition to the classification backbones already introduced, the experiments were also carried out with the EfficientNet [63] backbone.

#### 4.8.2. Network Architecture

Although the U-Net architecture delivers good results, the framework was tested with different network architectures (see Section 3.2). All three backbones were additionally evaluated with the FPN architecture. With this architecture, very similar results were achieved (see Table 7).

Experiments with more complex architectures like DeepLabv3+ did not show any improvements. Due to the small amount of training data, overfitting quickly occurred.

#### 4.8.3. Clustering Hyperparameters

**Figure 9:**
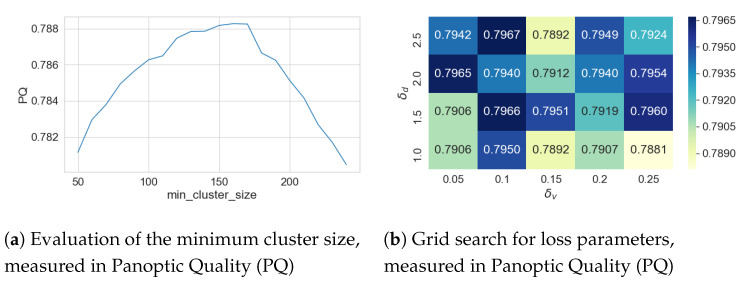
(**a**) Evaluation of the minimum cluster size (on the reduced 320 × 256 px images) showing a stable interval in the range 80 to 200; (**b**) Panoptic Quality (PQ) for different thresholds $\delta_{v}$ and $\delta_{d}$ which control the distances in the discriminative loss.

To optimize the density-based clustering, the thresholds $\delta_{v}$ and $\delta_{d}$ in the discriminative loss are available as hyperparameters (see Section 3.5). They control how close the clusters are moved together or how much distance different clusters have to keep from each other. As shown in Figure 5, the two parameters must actually only be sensibly adjusted to each other. Scaling the parameters ultimately only leads to a scaling of the embedding space and does not change the separation of the clusters.This assumption is supported by a grid search with $\delta_{v}\in \left[0.05,0.25\right]$ and $\delta_{d}\in \left[1.0,2.5\right]$ showing PQ values around 0.7931 with a standard deviation of σ=0.0027 (see Figure 9b).

**Figure A1:**
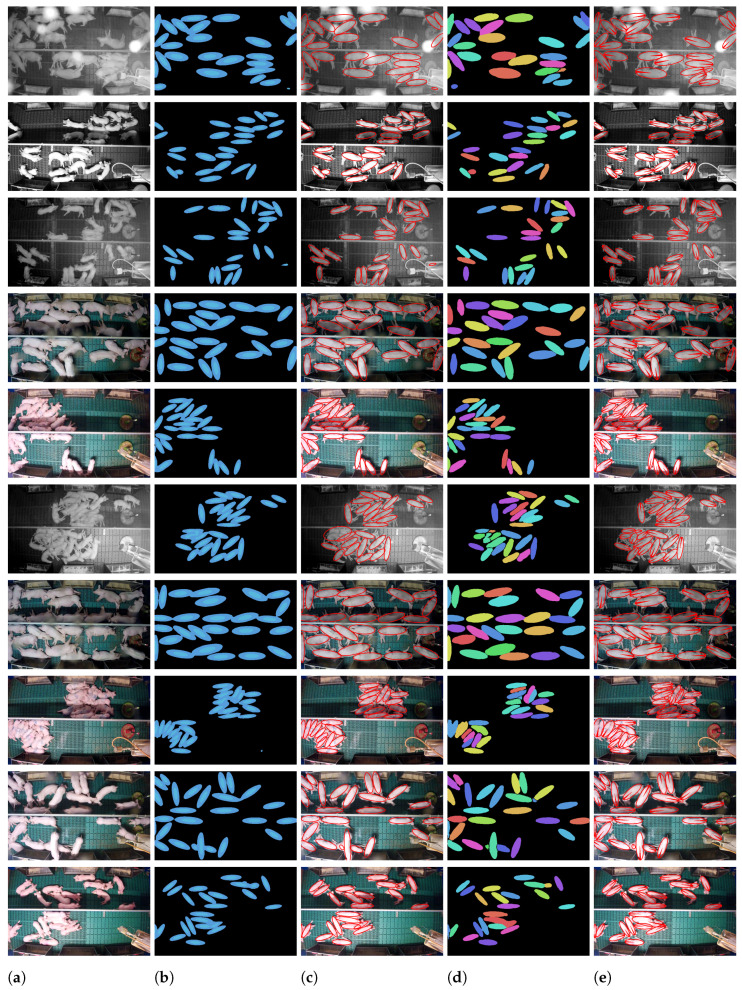
Examples of the results on images from the test set: The pictures show from left to right (**a**) the original image; (**b**) the prediction of the categorical segmentation; (**c**) the ellipses extracted from the categorical segmentation; (**d**) the prediction of the combined segmentation; and (**e**) the ellipses extracted from the combined segmentation (including orientation recognition). The filled part of the ellipses shows the identified orientation of the animals.

There is also the minimal cluster size, which refers to the number of pixels that at least belong to one pig. This parameter is therefore primarily dependent on the resolution of the input images and the size of the pigs and can only be set to a limited extent as a hyperparameter. As this is a minimum value, it must be chosen according to the size of the smallest animals in the processed data. As shown in Figure 9a, the value is stable in a large interval. Since HDBSCAN also takes outliers and noise into account, the minimum cluster size in this interval only affects the membership of individual pixels. Clusters are incorrectly split or merged only if the deviation from the stable interval is large. For the data set used here, a value of 100 has been proven to be reasonable, since the animals were observed over a longer period of time and therefore appear in different ages and sizes (see Figure A1).

## 5. Discussion

**Figure 10:**
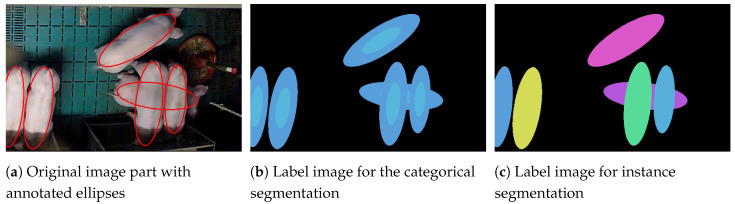
Example of the fragility of the categorical segmentation in case of strong overlaps. If the center of the animals is not visible, the segmentation cannot provide meaningful information about the hidden animal (**b**). The instance segmentation, on the other hand, does not have this problem (**c**).

**Figure 11:**
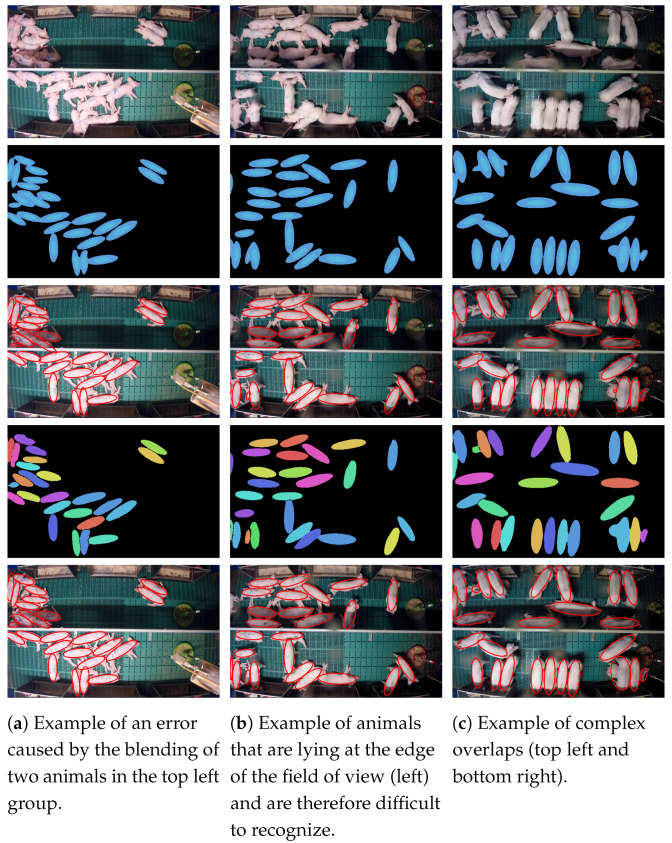
Examples of the difficulties that the data set contains: The pictures show from top to bottom the original image, the prediction of the categorical segmentation, the ellipses extracted from the categorical segmentation, the prediction of the combined segmentation, and the ellipses extracted from the combined segmentation.

As shown in Table 3, the quality of the extracted ellipses of the categorical segmentation and that of the combined approach are comparable on average. For more complex overlaps, in particular, the categorical segmentation theoretically reaches its limits when the core part of the pig is hardly visible (see Figure 10b). In such situations, pixel embedding should have shown its strengths but these situations hardly seem to occur in the actual data set. Therefore, the network was not able to learn these cases and produces correspondingly bad results (see Figure 11c). More visual results can be found in the Appendix A.

The cross-validation, the different backbones, and the architectures all deliver approximately the same results (see Table 6 and Table 7). This indicates a certain robustness of the presented method. However, the small amount of training data is problematic, as the more complex backbones or architectures cannot be used effectively due to the lack of variance in the images.

The choice of the PQ as evaluation metric makes sense with the methods presented, since the exact evaluation of the *intersection over union* provides information about how precisely the pixel accurate segmentation works. Unfortunately, this novel metric does not allow a direct comparison to other works. However, in order to allow a rough comparison, classical metrics like precision and recall are listed in Table 8. The authors of the only paper with a publicly accessible data set [20] give 91% precision and 67% recall for their test set. With our methods on our data set, we achieve values around 95% for both metrics. However, it should be noted that, although the test data in our data set comes from a different camera, the images in the test set do not differ fundamentally from the images in the training set. In [20], the images in the test set seem to deviate more from the training data. A direct comparison on the data set from [20] was not possible because ellipses cannot be reconstructed easily from the given key points. To our knowledge, other public data sets of pigs do not exist.

In general, the correct evaluation is difficult because there is no defined set of rules for annotation. In [20], for example, the pigs that are in the field of view but not in the observed bay were not annotated. A network that recognizes these pigs anyway would be punished with *false positives* here. Furthermore, there are also borderline cases in our data set where pigs are hardly visible but still marked by the human annotators. If such pigs are not found due to sanity checks like a minimum pixel number in clustering or a bad segmentation, false negatives are counted (see Figure 11b). Here, a publicly accessible data set with fixed rules would be useful in the future.

## 6. Conclusions

The methods shown here have achieved good results on the data used and offer a pixel accurate segmentation of the animals instead of bounding boxes or keypoints (see Figure A1). A key advantage over the existing methods is that more information can be extracted from the segmentation, e.g., conclusions can be drawn about the volume and thus the weight of the animals. Weight gain and other health factors can thus be determined and evaluated.

## Figures and Tables

**Table 1:** Data set statistic for the 1000 randomly selected and annotated images: The images of the test set are taken from a different camera than the images of the training and validation set.

Data Set	Total	Daylight	Night Vision
Train	606	361	245
Validation	168	96	72
Test	226	108	118

**Table 2:** Accuracy results of the binary segmentation experiment (measured with the Jaccard index): The experiment was carried out on all test images and separately on the daylight (D) and night vision (N) images only. Best results in bold.

Backbone	Acc	Acc (D)	Acc (N)
ResNet34	0.9730	0.9771	0.9692
Incep.-RN-v2	**0.9735**	**0.9774**	**0.9699**

**Table 3:** Detection results for the ellipses extracted with categorical segmentation and the combined segmentation: Regardless of the selected backbone, detection rates of about 95% (F1 score) are achieved. For detailed information about precision and recall, see Table 8. It is noticeable that, with the combined segmentation approach, the accuracy of the binary segmentation remains unaffected, although the segmentation head and the pixel-embedding head jointly influence the weights in the backbone. The experiments were carried out on all test images and separately on the daylight (D) and night vision (N) images only.

Categorical	PQ	PQ (D)	PQ (N)	F1	F1 (D)	F1 (N)	Cat. Acc	Cat. Acc (D)	Cat. Acc (N)
ResNet34	0.7920	0.8124	0.7738	**0.9550**	**0.9619**	**0.9487**	0.9612	0.9664	0.9565
Incep.-RN-v2	0.7943	0.8165	0.7742	0.9541	0.9614	0.9475	0.9612	0.9664	0.9564
**Combined**	**PQ**	**PQ (D)**	**PQ (N)**	**F1**	**F1 (D)**	**F1 (N)**	**Bin. Acc**	**Bin. Acc (D)**	**Bin. Acc (N)**
ResNet34	**0.7966**	**0.8181**	**0.7774**	0.9513	0.9588	0.9446	**0.9722**	**0.9761**	**0.9687**
Incep.-RN-v2	0.7921	0.8179	0.7689	0.9481	0.9566	0.9404	0.9707	0.9752	0.9666

**Table 4:** Results of orientation recognition: The network can correctly recognize orientation in 94% of the correctly found animals (true positive).

Backbone	Orien. Acc	PQ	Cat. Acc
ResNet34	**0.9428**	**0.7958**	**0.9644**
Incep.-RN-v2	0.9226	0.7898	0.9601

**Table 5:** Runtime evaluation of the proposed experiments for categorical segmentation and combined instance segmentation: All values are given as mean runtime and standard deviation over the 226 images of the test set.

Categorical	Network Inference (ms)	Postprocessing (ms)	
ResNet34	32.47±0.31	2.47±0.22
Incep.-RN-v2	88.91±0.23		
**Combined**	**Network Inference (ms)**	**Postprocessing (incl. Clustering) (ms)**	**Clustering (ms)**
ResNet34	41.98±1.12	2115.73±448.26	2068.53±443.72
Incep.-RN-v2	101.39±2.41		

**Table 6:** Results for a five-fold cross-validation on the data set: In each row, one of the cameras was declared as the test set, and the images from the four remaining cameras were use for training and validation.

Test Set	PQ	F1	Precision	Recall
Camera 1	0.7849	0.9571	0.9566	0.9575
Camera 2	0.7976	0.9602	0.9616	0.9588
Camera 3	0.7966	0.9513	0.9544	0.9482
Camera 4	0.7653	0.9344	0.9357	0.9331
Camera 5	0.8002	0.9544	0.9540	0.9548
Average	0.7889	0.9515	0.9525	0.9505
STD	0.0129	0.0090	0.0088	0.0094

**Table 7:** Results of the ablation study with the combined segmentation on the test data set: For this evaluation, a reduced image resolution of 320 × 256 pixels was used. The results highlight the marginal impact of the different architecture choices (U-Net vs. Feature Pyramid Network (FPN)) as well as the different backbones.

Combined U-Net	PQ	F1	Precision	Recall	Cat. Acc
ResNet34	0.7863	0.9457	0.9559	0.9358	0.9694
Incep.-RN-v2	0.7685	0.9326	0.9501	0.9157	0.9674
EfficientNet-B5	0.7768	0.9404	0.9471	0.9337	0.9692
**Combined FPN**	**PQ**	**F1**	**Precision**	**Recall**	**Cat. Acc**
ResNet34	0.7824	0.9442	0.9556	0.9332	0.9709
Incep.-RN-v2	0.7784	0.9414	0.9511	0.9319	0.9700
EfficientNet-B5	0.7861	0.9451	0.9556	0.9347	0.9709

**Table 8:** Detailed listing of F1 score, precision, and recall: The experiment was carried out on all test images and separately on the daylight (D) and night vision (N) images only.

Categorical	F1	F1 (D)	F1 (N)	Prec	Prec (D)	Prec (N)	Recall	Recall (D)	Recall (N)
ResNet34	**0.9550**	**0.9619**	**0.9487**	**0.9586**	**0.9678**	0.9503	**0.9514**	0.9560	**0.9472**
Incep.-RN-v2	0.9541	0.9614	0.9475	0.9577	0.9626	**0.9532**	0.9505	**0.9601**	0.9418
**Combined**	**F1**	**F1 (D)**	**F1 (N)**	**Prec**	**Prec (D)**	**Prec (N)**	**Recall**	**Recall (D)**	**Recall (N)**
ResNet34	0.9513	0.9588	0.9446	0.9544	0.9645	0.9454	0.9482	0.9531	0.9438
Incep.-RN-v2	0.9481	0.9566	0.9404	0.9495	0.9598	0.9402	0.9466	0.9535	0.9405
